# A Multimodal Conversational Chatbot for Emotional Support and Daily Assistance in Older Adults: Design, Development and Pilot Usability Evaluation

**DOI:** 10.3390/healthcare14131946

**Published:** 2026-07-01

**Authors:** Gema Parra-Cabrera, Michel Rodríguez-Fariñas, Antonia Rodríguez-Martínez, Francisco Daniel Pérez-Cano

**Affiliations:** 1 Departamento de Ingeniería de Sistemas Informáticos, Escuela Técnica Superior de Ingeniería de Sistemas Informáticos, Universidad Politécnica de Madrid, 28031 Madrid, Spain; gema.parra@upm.es; 2 Departamento de Informática, Escuela Politécnica Superior de Jaén, Universidad de Jaén, 23071 Jaén, Spain; mrf00035@red.ujaen.es (M.R.-F.); fdperez@ujaen.es (F.D.P.-C.); 3 Departamento de Psicología, Facultad de Trabajo Social, Universidad de Jaén, 23071 Jaén, Spain

**Keywords:** conversational chatbot, older adults, multimodal interaction, emotional support, conversational AI, human–computer interaction, healthy ageing, digital inclusion

## Abstract

**Background/Objectives**: Population ageing and the increasing prevalence of loneliness and social isolation represent major public health challenges. Digital health technologies, including conversational agents, have emerged as potential tools to support emotional well-being and daily functioning in older adults. This study presents the design and exploratory pilot evaluation of UjaBienestar, a multimodal conversational chatbot aimed at providing accessible emotional and practical support. **Methods**: A web-based multimodal system integrating text and voice interaction was developed using a Django backend and a Rasa-based conversational engine. The system was designed following user-centred and accessibility-oriented principles for older adults. A pilot usability and feasibility study was conducted with 10 participants using task-based interaction, observational data and pre/post-questionnaires. The exploratory evaluation focused on preliminary user perception, accessibility, and interaction feasibility rather than on statistically generalizable or clinically validated outcomes. **Results**: Participants reported high levels of perceived usability, accessibility, and acceptability. Multimodal interaction, particularly voice support, was positively valued. Users reported subjective perceptions of companionship during interaction within this pilot context. Initial barriers were mainly related to onboarding and first-time use. **Conclusions**: The findings suggest that the proposed system is a feasible and acceptable digital health support tool for older adults. While the results are preliminary, they highlight the potential of multimodal conversational technologies for supporting perceived emotional well-being, accessibility, and daily assistance in ageing populations. These findings are based on perceived user responses and do not represent clinically validated outcomes. Further large-scale and longitudinal studies are required to assess clinical and psychosocial impact.

## 1. Introduction

Population ageing has become one of the major social and healthcare challenges of the twenty-first century [1,2,3]. According to the United Nations, by 2050, more than 22% of the global population will be 60 years of age or older, nearly doubling current figures. In Europe, older adults already represent a substantial proportion of the population, a trend that is expected to continue in the coming decades, increasingly placing pressure on healthcare systems, social services, and community support structures. This demographic transformation not only poses economic and organisational challenges but also highlights the need to promote autonomy, well-being, and quality of life among older adults.

Among the most pressing issues associated with ageing are unwanted loneliness and social isolation. Holt-Lunstad et al. [4] identified social isolation as a significant risk factor for mortality, while [5] highlighted its broader impact on older adults’ health and well-being. More recently, Roy et al. [6] reported associations between loneliness, anxiety, depression, cognitive decline, and reduced functional capacity. At the same time, the persistent digital divide continues to affect older adults, limiting their access to digital services, communication tools, and technology-mediated support systems. Friemel [7] emphasized age-related inequalities in technology adoption, while Nimrod [8] discussed the consequences of digital exclusion for older adults’ social participation and access to support resources.

In this context, conversational agents and chatbot-based systems have emerged as promising technologies capable of providing accessible and scalable support. Montenegro et al. [9] reviewed the increasing use of conversational agents in healthcare settings, while Følstad and Skjuve [10] highlighted their potential to improve user engagement and service accessibility. More recently, Abdalrazaq et al. [11] reported encouraging evidence regarding the effectiveness and acceptability of conversational agents for health-related interventions. Recent advances in artificial intelligence, natural language processing, and multimodal interaction have enabled the development of intelligent systems that can assist users in healthcare, emotional well-being and daily task management.

Despite these advances, an important research gap remains from an interdisciplinary perspective that combines computer science, human–computer interaction, and social support technologies. Existing approaches often focus on clinical monitoring or generic task assistance, whereas fewer studies have explored the design of empathic, accessible, and multimodal systems aimed at reducing loneliness and improving everyday support in ageing populations.

Unlike previous studies primarily focused on clinical monitoring or general-purpose conversational assistants, this work proposes an integrated multimodal framework explicitly designed to support emotional well-being, daily assistance, and digital inclusion in older adults. The proposed system integrates text and voice interaction, reminder functionalities, and user-centred design principles specifically adapted to the characteristics of older users. A preliminary pilot evaluation was conducted to assess usability, feasibility, and perceived usefulness in a real-user context.

In addition, it is important to recognize that many existing digital health solutions targeting older adults are often developed without sufficient consideration of emotional and social dimensions of interaction. While technological advances have enabled increasingly sophisticated systems, their adoption in ageing populations remains strongly dependent on perceived trust, emotional connection, and usability. Therefore, the integration of emotional support mechanisms within assistive technologies should not be considered an optional feature, but rather a central design requirement for promoting meaningful engagement and long-term use.

While the proposed system does not introduce a novel conversational AI model or machine learning architecture, its contribution lies in the integration of multimodal accessibility, empathic conversational design, and daily assistance functionalities into a unified framework specifically tailored to older adults. In contrast to general-purpose conversational assistants, the proposed approach prioritizes accessibility-oriented interaction, emotional support perception, and ageing-related usability requirements within a single conversational environment. The main contributions of this work are threefold: (i) the design and implementation of a multimodal conversational system tailored to older adults, (ii) the integration of accessibility and empathic conversational design principles, and (iii) the preliminary validation of its feasibility and usability as an assistive technology solution for healthy ageing and social inclusion.

## 2. Related Work

Conversational agents have increasingly been explored within digital health and assistive technology contexts due to their potential to provide scalable and continuously available support [9,10]. Recent advances in conversational AI and multimodal interaction have enabled the development of systems for healthcare support, emotional well-being, and daily assistance [9,11]. In particular, conversational systems have demonstrated promising potential in emotionally sensitive contexts such as loneliness support and psychological well-being [11,12].

Within ageing populations, conversational technologies have attracted growing attention as potential tools to support emotional well-being, accessibility, and daily assistance. Older adults frequently encounter barriers related to digital literacy, usability, and social isolation, which may negatively affect technology adoption and emotional well-being [7,8]. Consequently, recent research has increasingly explored conversational and assistive technologies capable of supporting accessibility, companionship, and everyday interaction in older adults [11,12].

Previous studies involving conversational systems for older adults have highlighted usability, accessibility, and perceived usefulness as central factors influencing technology acceptance and sustained interaction [13,14,15]. In addition, conversational naturalness, perceived empathy and emotional responsiveness have been identified as relevant design dimensions for improving engagement and perceived companionship in emotionally sensitive contexts [11,12].

Several studies have explored the use of intelligent assistants and conversational systems specifically for older adults. Martin-Hammond et al. [13] reported that older adults positively value intelligent assistants for health-related information management, although concerns regarding trust, usability, privacy, and data security remain significant. Similarly, Liu et al. [14] found that usability, emotional connection, perceived usefulness and ease of interaction strongly influence older adults’ intention to adopt voice assistants.

Recent research has increasingly focused on conversational systems as tools to alleviate loneliness and enhance emotional well-being in ageing populations. Rodríguez-Martínez et al. [16] conducted a qualitative analysis, which showed that chatbots may serve as meaningful emotional support tools for older adults experiencing loneliness. Their findings suggest that conversational systems may provide a perceived sense of companionship, emotional comfort and support in daily life situations.

In the same line, Loveys et al. [17] demonstrated the feasibility of digital human conversational interventions for reducing loneliness and stress in both younger and older adults. Their results support the idea that intelligent conversational systems may become relevant tools for emotional support and social technology interventions.

Furthermore, the integration of voice interaction has been identified as particularly relevant for older users, as it may reduce interaction barriers related to visual impairments, reduced motor skills and low digital literacy [7,18]. Voice-based interfaces may offer a more natural and less cognitively demanding interaction model compared with traditional graphical interfaces, especially in older populations.

From the perspective of conversational experience, previous studies have also highlighted the gap between user expectations and actual system capabilities. Luger and Sellen [19] showed that users frequently expect conversational agents to demonstrate greater contextual awareness, flexibility, and empathic understanding than most current systems can realistically provide. This gap is especially relevant in emotionally sensitive contexts such as loneliness support and social companionship.

Recent years have also seen the emergence of both commercial voice assistants and research-oriented conversational systems aimed at emotional support, daily assistance, and social interaction. Commercial voice assistants, including platforms such as Alexa and Google Assistant, have increasingly been explored as accessible interaction technologies for older adults and health-related support contexts due to their voice-based interaction capabilities and potential to reduce usability barriers associated with traditional interfaces [13,20]. In parallel, conversational systems have been focused on emotional companionship and mental health support, such as those of Replika [21] and Woebot [22], and socially assistive technologies in elderly care contexts [23] have incorporated emotionally oriented dialogue and supportive interaction strategies. However, these systems differ considerably in terms of accessibility-oriented design, multimodal interaction strategies, and explicit adaptation to the needs of older adults. In many cases, existing solutions focus either on emotional companionship, mental health support, or task assistance independently, while fewer approaches attempt to integrate these dimensions within a unified conversational framework specifically tailored to ageing populations.

As shown in Table 1, existing systems often provide isolated functionalities related to voice interaction, emotional support, or task assistance. In contrast, the proposed framework seeks to integrate multimodal accessibility, empathic conversational interaction, and daily assistance functionalities within a unified conversational environment specifically tailored to older adults.

Therefore, there is still a significant gap in the design and evaluation of conversational systems specifically tailored to support older adults through empathic interaction, voice-enabled accessibility, and daily assistance functionalities. This study addresses this gap by proposing a multimodal conversational chatbot that integrates emotional support, reminder management, and accessible interaction principles specifically designed for older adults.

## 3. Materials and Methods

This study adopted a mixed-methods exploratory design that combines the development of a multimodal conversational assistant with a preliminary pilot usability evaluation involving older adults. The study workflow included four main stages: (i) needs analysis and system design, (ii) technical development and integration, (iii) pilot usability evaluation, and (iv) qualitative analysis of user feedback.

### 3.1. Study Design and Development Workflow

This study was designed as a pilot feasibility and usability study aimed at exploring initial system acceptability, interaction patterns and perceived usefulness, rather than measuring clinical or long-term health outcomes.

The project followed an iterative development process inspired by user-centred design principles and agile prototyping strategies.

The initial stage consisted of a literature review focused on active ageing, social isolation, conversational technologies, and accessibility challenges in older adults. This phase informed the identification of functional and emotional support requirements for the target population.

Subsequently, the conversational flows and interface prototypes were designed and refined before implementation. The development process was structured into iterative cycles that incorporated early validation feedback in order to improve accessibility and conversational usability.

### 3.2. System Architecture

The proposed system, UjaBienestar, was implemented as a web-based multimodal conversational platform specifically designed for older adults.

The technical architecture follows a modular client–server model composed of the following components:

- Frontend interface: responsive web application optimized for desktop, tablet, and smartphone devices;
- Backend layer: Django-based server, a Python (3.9.13) web framework used for web application management, user sessions and data processing;
- Conversational engine: Rasa framework, an open source conversational AI platform used for intent recognition, dialogue management, and rule-based conversational flows;
- Temporal entity extraction: Duckling service, a natural language processing tool used to recognize dates, times, and temporal expressions from user messages;
- Speech processing: speech-to-text and text-to-speech modules;
- External services: Google OAuth 2.0, Google Calendar API, and Google People API;
- Database: persistent storage of reminders, interaction logs, and user sessions.

The use of Rasa enabled the combination of learned conversational flows through *stories.yml* and deterministic responses through *rules.yml*, providing both flexibility and control for critical interaction scenarios such as reminders and emergency-related actions.

**Figure 1:**
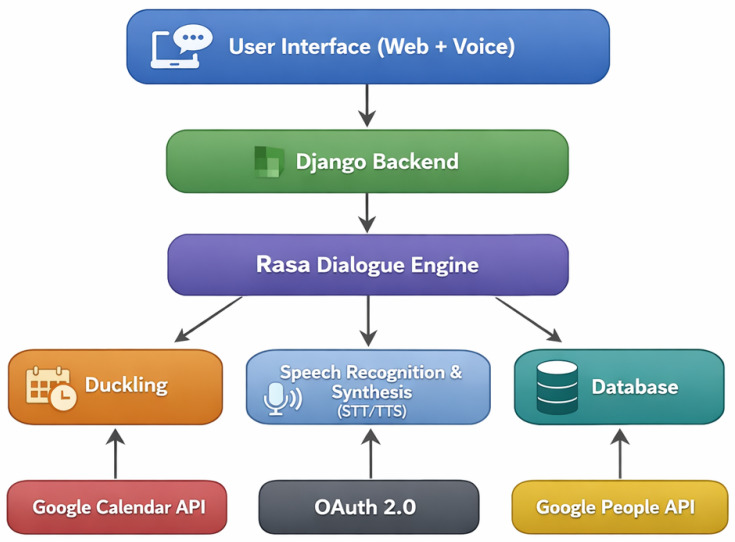
System architecture of the UjaBienestar multimodal conversational platform.

As illustrated in Figure 1, the proposed system follows a modular client–server architecture designed to support multimodal interaction and service integration.

The interaction begins at the user interface layer, where older adults can communicate through both text and voice input. These requests are processed by the Django backend, which manages user sessions, authentication, business logic, and communication with the conversational modules.

At the core of the system, the Rasa dialogue engine is responsible for intent recognition, dialogue state tracking and response generation. This module combines predefined rule-based conversational flows with story-driven dialogue management to ensure both flexibility and control in critical interaction scenarios.

The conversational engine interacts with three specialized modules. First, the Duckling temporal processing component extracts time- and date-related entities required for reminders and appointment scheduling. Second, the voice processing module manages speech-to-text and text-to-speech functionalities, enabling accessible multimodal interaction. Third, the persistent storage layer stores user sessions, reminders, interaction logs, and selected user preferences.

Finally, the architecture integrates several external services. Google OAuth 2.0 is used for secure authentication and user session management, Google Calendar API supports appointment and reminder synchronization, and the Google People API enables access to emergency contact information and contact-based functionalities.

This modular design facilitates scalability, maintainability, and future integration of adaptive and personalized conversational capabilities.

A detailed description of the technical architecture is provided to ensure transparency, reproducibility, and clarity regarding the implementation of the proposed digital health support system.

### 3.3. Conversational Framework and Accessibility Design

The conversational system was designed following a user-centred and accessibility-oriented approach, with particular emphasis on empathic interaction as a core design principle rather than a purely stylistic feature.

Empathic conversational design was operationalized through several concrete strategies integrated into the dialogue system. The selection of these strategies was informed by the previous literature on conversational agents for emotional support and digital mental health. Empathy was therefore operationalized as a design principle through emotional validation, supportive follow-up questions, and non-judgmental language rather than through formal computational empathy modelling or standardized empathy assessment metrics. First, the chatbot incorporates emotionally aware prompts that acknowledge user input and provide validation, such as expressions of understanding (“I understand how you feel”) and supportive follow-ups (“Would you like me to help you with something?”). Second, the system uses guided conversational structures that reduce cognitive load by offering predefined response options and step-by-step interaction flows. Third, confirmation strategies are systematically applied in critical actions (e.g., reminders or emergency-related interactions) to ensure clarity and user confidence.

The conversational framework was organized around predefined rule-based interaction flows covering greeting and onboarding, emotional support conversations, reminder and medication management, emergency assistance procedures, and session closure. These rules were designed to reduce cognitive load, maintain conversational predictability, and provide confirmation-based interaction for critical actions. The conversational structure was intentionally simplified to accommodate varying levels of digital literacy and age-related usability requirements. The selection of these interaction patterns was informed by the requirement analysis phase and by accessibility-oriented design recommendations reported in the literature for older adult populations.

**Figure 2:**
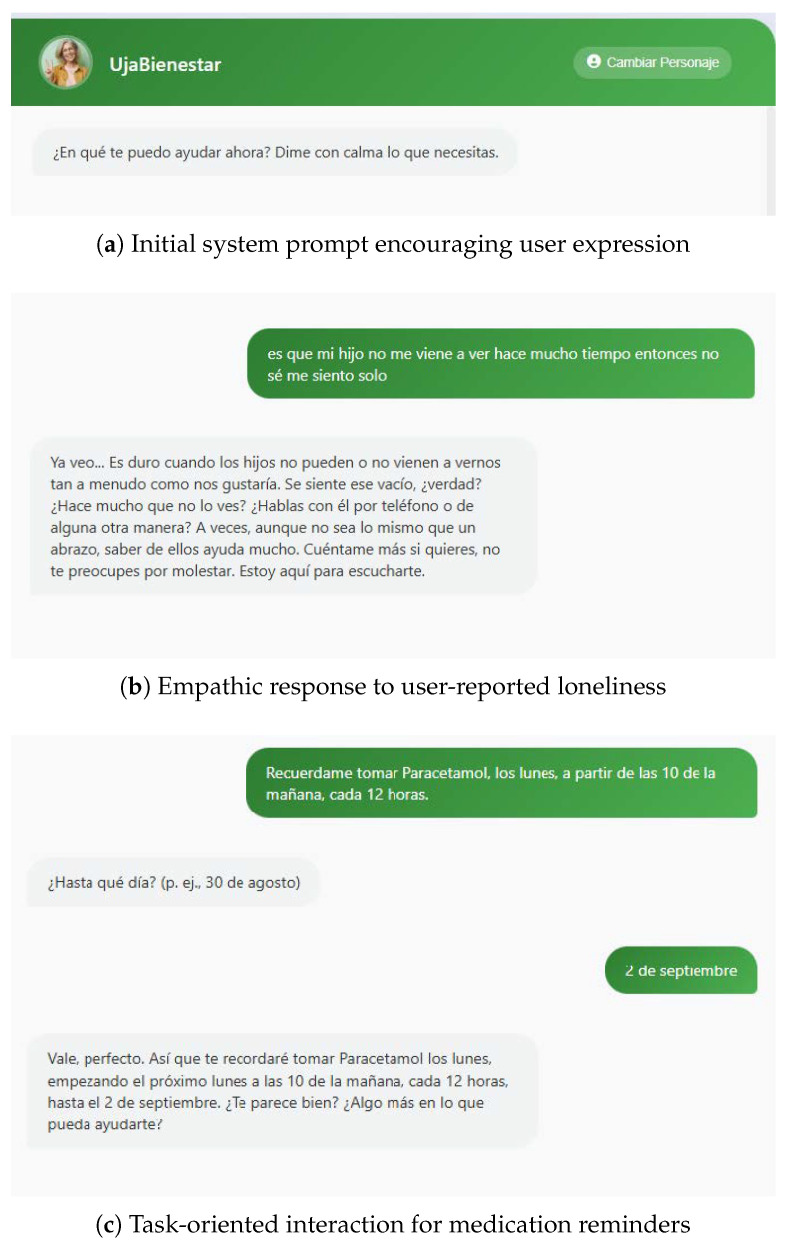
Examples of conversational interaction in the UjaBienestar system, illustrating empathic and task-oriented dialogue strategies (all interaction examples are presented in Spanish; English translations are provided in Appendix A).

As illustrated in Figure 2, the conversational system integrates both empathic and task-oriented dialogue strategies. The examples show how the chatbot encourages user expression, provides emotionally supportive responses through validation and follow-up questions, and assists in practical daily tasks such as medication reminders. More specifically, Figure 2a presents the initial system prompt designed to encourage user engagement through a calm and accessible tone. Figure 2b illustrates an empathic response to explicitly expressed emotional discomfort and loneliness-related conversational cues, where the system acknowledges the emotional context and provides supportive and guiding dialogue. Finally, Figure 2c shows a task-oriented interaction related to medication reminders, including clarification and confirmation steps to ensure accuracy.

Additionally, the system avoids directive or overly technical language, prioritizing a warm, reassuring, and non-judgmental tone instead. This design choice aims to foster trust and perceived companionship, which are particularly relevant in contexts of loneliness and social isolation.

From an accessibility perspective, the interface integrates large typography, simplified navigation, multimodal interaction (text and voice), and clear conversational layouts. Voice interaction was incorporated as a key accessibility feature aimed at facilitating interaction for users with varying levels of digital literacy and physical or cognitive limitations.

Regarding emergency assistance, the system incorporates a structured interaction flow that prioritizes user safety and clarity. Upon detecting an emergency-related intent, the chatbot activates a confirmation-based sequence to avoid unintended actions. Once confirmed, the system facilitates rapid access to predefined contacts, ensuring that critical communication can be established efficiently. This design balances responsiveness with control, minimizing the risk of false activations while maintaining accessibility.

**Figure 3:**
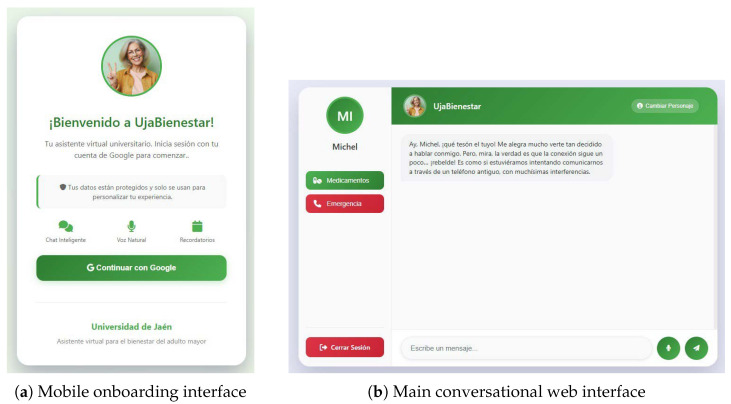
Main user-facing views of the UjaBienestar platform, including the onboarding interface and the primary conversational environment (all interaction examples are presented in Spanish; English translations are provided in Appendix A).

As illustrated in Figure 3, the proposed interface was specifically designed to maximize accessibility and ease of interaction through simplified visual elements, a clear conversational layout, and multimodal communication channels. The onboarding interface (Figure 3a) supports initial user engagement through guided interaction, while the main conversational interface (Figure 3b) provides a structured environment for continuous dialogue and task execution.

Complementary functionalities also include voice personalization, enabling users to select preferred speech characteristics and adapt the auditory interaction experience to individual preferences.

Importantly, empathic interaction in this system is not limited to linguistic tone, but is structurally embedded within the dialogue design. The combination of emotional validation, guided responses, and confirmation strategies allows the system to maintain conversational coherence while supporting user expression. This structured approach to empathic interaction distinguishes the system from purely reactive conversational agents and contributes to a more supportive and engaging user experience.

These design elements collectively differentiate the system from standard task-oriented chatbots by explicitly integrating emotional support mechanisms, accessibility features, and structured daily assistance within a unified conversational framework tailored to older adults.

### 3.4. Participants and Pilot Evaluation Procedure

A pilot usability and feasibility evaluation was conducted in a controlled environment with 10 older adult participants (mean age = 70 years), including 6 women and 4 men ranging from basic smartphone users to participants with more regular use of digital applications.

Participants presented heterogeneous levels of digital literacy, ranging from basic smartphone users to individuals with more frequent use of digital applications. This variability was intentionally considered to reflect realistic usage conditions among older adult populations. No participants reported prior extensive experience with conversational agents.

Participants were recruited through convenience sampling from local community and senior social engagement networks. The use of convenience sampling was considered appropriate for this early-stage exploratory pilot evaluation, where the primary objective was to obtain preliminary usability and interaction feedback rather than statistically representative evidence. All participants were autonomous older adults without severe cognitive impairments and provided informed consent prior to participation.

Participants interacted freely with the conversational assistant during sessions lasting approximately 25–30 min, with an average interaction time of approximately 27 min per participant. Interactions typically included conversational exchanges related to emotional support, daily routines, reminder configuration, and companionship-oriented dialogue.

The evaluation protocol was structured to ensure consistency across participants while allowing natural interaction with the system. Each session followed a predefined sequence that combined guided tasks and free interaction periods. Participants were encouraged to explore the system beyond the predefined tasks in order to capture spontaneous usage behaviours and natural conversational dynamics. Researchers adopted a non-intrusive observation approach, intervening only when participants encountered significant difficulties that prevented task completion.

During the sessions, researchers remained physically present in the testing environment but avoided actively guiding participant interaction unless technical or interaction difficulties prevented task continuation. Participants were informed that assistance was available if needed, although researchers minimized verbal intervention in order to reduce potential influence on conversational behaviour and usability perception.

Participants were asked to complete a set of representative tasks designed to simulate real-world use cases: logging into the platform using a test Google account, initiating a free empathic conversation, using voice-based interaction, creating reminders and appointments, checking scheduled events, and navigating the interface.

During the sessions, researchers collected direct observational notes regarding task completion, interaction barriers, time to completion, hesitation points, and spontaneous user reactions.

In addition, two structured questionnaires were administered:

- Pre-use questionnaire: demographic and technological profile, previous experience, expectations, and perceived initial barriers.
- Post-use questionnaire: usability, accessibility, overall satisfaction, and emotional perception of the interaction.

The questionnaires were specifically designed for this pilot study to capture usability perception, accessibility, and emotional response during interaction. Given the exploratory and early-stage nature of this pilot study, the evaluation focused on identifying usability patterns, accessibility barriers, and perceived interaction experiences rather than obtaining statistically representative or clinically validated outcomes.

The questionnaires were developed specifically for this exploratory pilot evaluation based on the study objectives and the dimensions identified in previous research related to usability, accessibility, and emotional perception in conversational systems for older adults. The instruments were not intended as psychometrically validated assessment tools, but rather as exploratory user perception instruments aimed at capturing preliminary interaction experiences and usability feedback.

This mixed collection strategy enabled triangulation between observed behaviour and self-reported perceptions.

### 3.5. Ethical and Privacy Considerations

The system was developed following privacy-by-design and ethical AI principles to ensure secure and responsible interaction with users. Secure authentication mechanisms based on OAuth 2.0 were implemented, while communication and stored information were protected through encrypted data transfer and storage procedures. In addition, the chatbot was conceived exclusively as a supportive conversational tool and not as a replacement for professional, clinical, or human care, particularly in emotionally sensitive situations.

The pilot evaluation was designed as a non-invasive usability study focused on observing participants’ interaction with the conversational system in a natural and non-interventional context. No clinical procedures, biomedical experimentation, medical interventions, or sensitive biomedical data collection was carried out during the study.

Before participation, all users received a clear explanation of the study objectives, interaction procedures, and data-handling conditions and informed consent was obtained from all participants. To preserve privacy and confidentiality, all collected information and interaction records were anonymized using coded identifiers prior to analysis, preventing the direct association of data with individual participants.

Furthermore, the study was conducted with special attention to respectful, empathetic, and user-centred interaction, ensuring that participants’ experiences and perspectives were represented responsibly throughout the evaluation and reporting process.

### 3.6. Data Collection and Analysis

**Figure 4:**
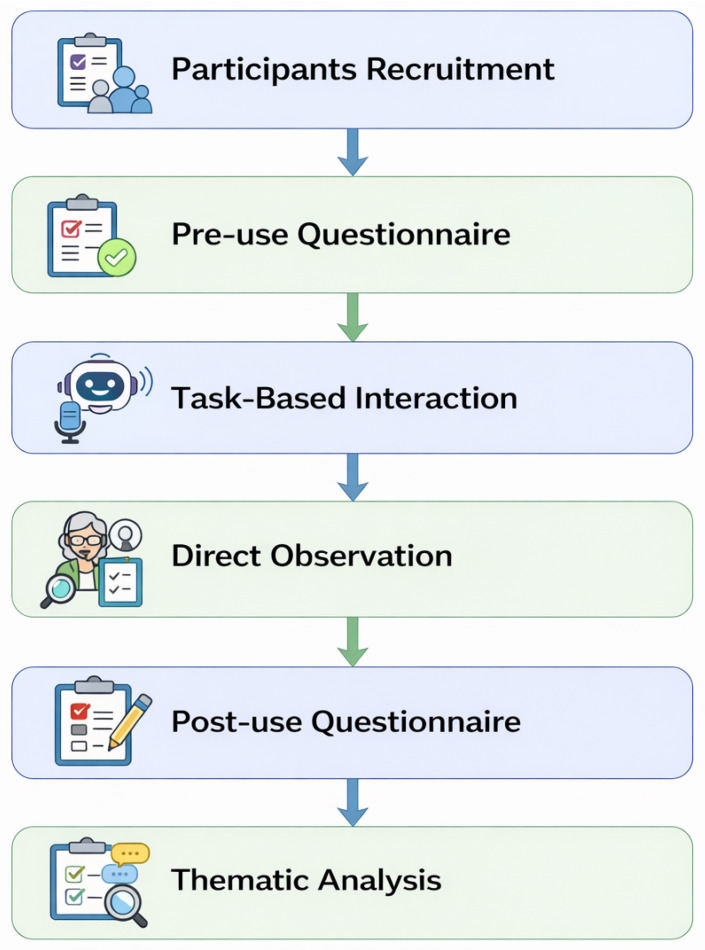
Workflow of the pilot usability evaluation, including participant recruitment, task-based interaction, data collection, and thematic analysis.

Following the workflow summarized in Figure 4, the data collection process combined three complementary sources: direct observational records during task execution, pre- and post-use questionnaires, and verbal qualitative feedback collected immediately after the session.

The collected data were analysed using a qualitative thematic analysis approach. Codes were iteratively generated from observational notes and participant feedback and subsequently grouped into predefined analytical dimensions, including usability and accessibility, emotional support perception, interaction barriers, and improvement opportunities.

The coding and thematic grouping process was conducted collaboratively by the research team through iterative discussion and refinement of the identified categories. Given the exploratory nature of the study, the analysis focused on identifying recurring patterns and meaningful user experiences rather than establishing formal inter-rater reliability metrics.

No standardized psychometric or clinical instruments were incorporated at this stage, as the primary objective was to evaluate preliminary usability, accessibility, and interaction feasibility. Future studies should incorporate validated quantitative measures related to usability, technology acceptance, loneliness, and emotional well-being.

This approach allowed the identification of system strengths, usability bottlenecks, and future design priorities.

The findings derived from this analysis should be interpreted as exploratory and descriptive, providing preliminary insights into system feasibility and user perception rather than statistically generalizable results.

## 4. Results

As expected in an exploratory pilot evaluation with a limited participant sample, the findings should be interpreted as preliminary descriptive insights into usability and interaction perception rather than statistically generalizable evidence.

Despite the limited sample size, the results reveal recurrent usability and interaction perception patterns within this pilot context.

The preliminary pilot evaluation provided relevant insights into the usability, accessibility and perceived usefulness of the proposed multimodal conversational chatbot. A summary of the main findings from the pilot evaluation is presented in Table 2, showing a highly positive perception of usability, multimodal interaction, and emotional support.

The quantitative frequencies reported in Table 2 are intended to descriptively summarize recurrent usability and interaction perceptions within the pilot sample rather than provide statistically inferential evidence.

### 4.1. Usability and Accessibility

Overall, participants reported a highly positive perception of the system in terms of usability and ease of interaction. As shown in Table 2, 9 out of 10 participants described the interface as easy to use and intuitive. The multimodal design, which combined text and voice interaction, was particularly valued as it allowed users to choose the communication modality that best suited their preferences and abilities. The accessible interface design, including large typography, simplified navigation, and guided interaction options, was perceived as beneficial for reducing technological barriers. This high level of agreement suggests that usability perception was not isolated but consistently experienced across users with different levels of digital literacy.

Several participants highlighted that the interface was easy to understand and that the interaction flow was perceived as clear and easy to follow. Voice interaction was identified as one of the most positively perceived functionalities. As summarized in Table 2, 9 out of 10 participants positively valued the possibility of interacting through speech. The possibility of receiving spoken responses contributed to a more natural and less demanding interaction experience, particularly among users with lower confidence in digital technologies.

### 4.2. Perceived Emotional Support

One of the most relevant findings of the pilot study was the positive perception of the chatbot as a support tool beyond purely functional assistance. A particularly relevant result was that all participants within this pilot sample (10/10) reported subjective perceptions of companionship and emotional accompaniment during interaction, as shown in Table 2, although this perception may be influenced by the novelty of the interaction and the controlled evaluation setting. The uniformity of this response across participants reinforces the relevance of empathic conversational strategies as a core component of user experience in this context. These perceptions should not be interpreted as evidence of clinically measurable emotional improvement.

Participants described the interaction as a form of accompaniment and emotional support, frequently emphasizing the feeling of being listened to. This perception was especially associated with the empathic tone of the conversational responses and the use of supportive prompts integrated into the dialogue structure.

The inclusion of emotionally oriented messages and confirmation strategies contributed to generating a more natural and reassuring interaction experience.

In fact, one participant explicitly stated that interacting with the system “felt like someone was listening”, illustrating the perceived sense of companionship generated during the interaction.

These findings reflect perceived emotional support during interaction rather than clinically validated emotional outcomes.

Importantly, this study does not provide objective or clinically validated evidence of emotional support effectiveness. The findings should be interpreted as subjective user perceptions within a controlled pilot context. Therefore, further research is required to assess whether such systems can produce measurable improvements in emotional well-being using validated psychological or clinical instruments.

This consistency across participants suggests that the perceived emotional support was not an isolated outcome but a recurrent interaction pattern.

These interaction patterns are further illustrated in Figure 2b, where the system provides emotional validation and supportive follow-up responses.

### 4.3. Interaction Barriers

Despite the generally positive feedback, some interaction barriers were identified during the pilot evaluation. Initial interaction barriers were reported by two participants, mainly during the onboarding phase and first contact with the voice-based functionalities, as summarized in Table 2. The relatively low frequency of reported barriers suggests that, despite initial onboarding challenges, the system was generally accessible after initial familiarization. In addition, minor limitations related to the rigidity of some conversational flows were observed when users introduced unexpected responses or deviated from the predefined dialogue paths. These issues suggest the need for greater conversational flexibility and improved onboarding support for first-time users.

To address these initial interaction barriers, future iterations of the system will incorporate guided onboarding mechanisms, including step-by-step conversational tutorials and progressive disclosure of functionalities.

### 4.4. Improvement Opportunities

Participants also provided several suggestions for future improvements. As presented in Table 2, 7 out of 10 participants suggested expanding the range of conversational topics and improving response naturalness. The most frequently mentioned proposals included incorporating music, news, and memory-related dialogues; improving the naturalness of the synthesized voice; and increasing the contextual adaptability of the responses. These findings provide valuable guidance for future iterations of the system and support the feasibility of continuing the development towards more adaptive and personalized conversational support solutions.

## 5. Discussion

The results of this preliminary exploratory study suggest that multimodal conversational systems may represent a promising preliminary approach for supporting older adults in terms of perceived emotional well-being, accessibility, and daily assistance. The positive perception of usability and the favourable response to both text- and voice-based interaction reinforce the relevance of designing assistive technologies specifically adapted to the needs of ageing populations.

Beyond the immediate findings, the results can be interpreted within the broader context of increasing interest in non-clinical digital interventions aimed at promoting well-being and social connectedness among older adults. While traditional healthcare approaches often focus on diagnosis and treatment, complementary digital solutions such as conversational systems may contribute to preventive and supportive everyday interaction by addressing emotional and social needs in everyday life.

From a broader assistive technology perspective, conversational support tools such as the proposed system may represent a potentially useful complementary resource for providing accessible emotional support and assistance in non-critical daily activities among older adults. Their continuous availability and multimodal accessibility suggest potential applicability within supportive ageing and independent living contexts, although further large-scale and longitudinal evaluation would be required to assess their real-world impact.

One of the most relevant findings concerns the perceived emotional support reported by participants during interaction. While reminder and task assistance functionalities are already present in widely adopted commercial conversational assistants, participants in this study frequently described the interaction as a form of companionship and emotional accompaniment. As illustrated in Figure 2, empathic conversational strategies such as emotional validation and supportive follow-up responses were integrated into the dialogue design. This perception appears particularly relevant in the context of older adults, where accessibility-oriented interaction, empathic conversational tone and multimodal communication may play an important role in technology acceptance and perceived usefulness.

However, it is important to distinguish between perceived emotional support and clinically measurable emotional outcomes. While participants reported a sense of companionship and emotional accompaniment, this study does not establish whether such interactions lead to measurable improvements in psychological well-being. This distinction highlights the need for future studies incorporating validated assessment instruments and longitudinal designs.

Beyond individual interaction, these findings are particularly relevant in the context of increasing societal concern regarding loneliness and social isolation in ageing populations. Technologies capable of providing accessible and emotionally supportive interaction may play a complementary role in addressing these challenges, especially in scenarios where human contact is limited or irregular.

This result is consistent with previous studies reporting the potential of conversational systems to alleviate loneliness and provide perceived emotional comfort among older adults [16,17]. In particular, the incorporation of empathic prompts and warm conversational language appears to have contributed positively to trust and engagement, in line with previous findings in human–computer interaction and digital mental health contexts [11,12].

The importance of voice interaction should also be highlighted. Participants positively valued the possibility of using speech as an alternative interaction modality, which is consistent with previous studies emphasizing the benefits of voice interfaces for older adults, particularly in the presence of reduced visual acuity, motor limitations, and low digital literacy [14,18]. These findings reinforce the relevance of multimodal interaction as a design strategy for improving accessibility and technology acceptance in ageing populations.

From a usability perspective, the results also support the importance of user-centred design principles such as simplified navigation, large typography, guided interaction flows, and confirmation-based dialogues. These elements are aligned with established usability frameworks [24,25] and appear particularly relevant in reducing cognitive load and facilitating adoption among first-time users.

Importantly, the objective of this study was not to benchmark a novel conversational AI model or compete with large-scale commercial conversational assistants, but rather to explore the feasibility of an integrated and accessibility-oriented conversational support framework for older adults. Consequently, the contribution should be interpreted primarily from a human–computer interaction and assistive technology perspective rather than from the perspective of conversational AI performance optimization.

However, the study also revealed several limitations and opportunities for improvement. In particular, the rigidity observed in some conversational flows indicates the need for more flexible dialogue management and greater contextual adaptability. This limitation has also been reported in previous research, where users frequently expect conversational agents to exhibit a higher degree of conversational awareness and empathy than what current systems typically provide [19].

From a broader perspective, this work contributes to the growing field of assistive conversational systems for healthy ageing by proposing an integrated framework that combines emotional support, reminder management, and accessible multimodal interaction. Unlike general-purpose commercial conversational assistants, the proposed system was specifically designed to address the emotional, accessibility and daily assistance needs of older adults through a user-centred conversational framework. However, no direct comparative evaluation with commercial systems was conducted in this study, and therefore, no performance claims are made. In contrast to previous approaches primarily focused on clinical monitoring or isolated functionalities, the proposed system seeks to address both the social and practical needs of older adults in daily life.

Nevertheless, several limitations must be acknowledged. First, the study was conducted as a preliminary pilot evaluation with a limited number of participants, which restricts the generalizability of the findings. Additionally, participants were recruited through community and senior social engagement networks, which may have introduced a selection bias toward older adults with higher levels of social participation and lower risk of severe loneliness or social isolation. Consequently, the findings may not fully represent the perceptions and interaction needs of more socially isolated or vulnerable older adult populations. This sampling characteristic may be particularly relevant when interpreting the perceived companionship and emotional support findings reported in this study. Older adults experiencing higher levels of loneliness, social isolation, or reduced social support may interact with or perceive conversational support systems differently from participants with active social engagement networks.

Importantly, the study does not aim to provide evidence of clinical or long-term impact, but rather to explore initial indications of user perception and system acceptability. Second, the evaluation relied primarily on qualitative feedback and exploratory usability observations, without the use of standardized clinical or psychometric instruments. Additionally, the supervised nature of the evaluation and the novelty of the system may have influenced participant responses, potentially introducing social desirability and novelty effects.

Furthermore, the current version of the chatbot relies on partially predefined conversational flows, which may limit contextual flexibility and adaptive response generation. This limitation suggests the need for more advanced dialogue management strategies capable of improving conversational naturalness and personalization.

In addition, the current version of the system primarily reacts to explicitly expressed conversational cues and user-reported emotional states rather than autonomously inferring complex emotional conditions such as loneliness from indirect behavioural or linguistic indicators. Future work should therefore explore more adaptive and personalized conversational approaches capable of identifying subtle indicators of emotional vulnerability and social isolation.

Additionally, no direct comparative evaluation against commercial conversational assistants or baseline chatbot systems was conducted. Future research should incorporate controlled comparative studies in order to better quantify the relative strengths and limitations of the proposed framework.

Future work should therefore include larger and more diverse samples in order to validate the findings across different ageing profiles and levels of digital literacy. In addition, future evaluations should incorporate standardized usability and psychosocial instruments such as the System Usability Scale (SUS), Technology Acceptance Model (TAM) measures, and validated loneliness or emotional well-being scales, together with larger and more diverse participant samples, in order to provide more robust quantitative evidence. Longitudinal studies are also required to assess sustained use of the system and its potential impact on emotional well-being, loneliness reduction, and digital inclusion.

Future research may also explore the integration of personalized memory, adaptive user profiles, and adaptive recommendation systems to enhance user experience and long-term engagement.

This study should therefore be understood as a system-oriented contribution with preliminary user validation rather than a validated digital health intervention study. This positioning is particularly relevant in early-stage digital health technologies, where system design and usability validation constitute necessary steps prior to large-scale clinical evaluation.

The findings support the potential of multimodal conversational systems as assistive technologies for older adults and provide preliminary directions for future research in adaptive, empathic, and accessible conversational support systems. Future developments should explore larger and more diverse participant samples, including older adults experiencing higher levels of social isolation or reduced social support. Additional research should incorporate standardized usability, technology acceptance, and psychosocial assessment instruments to better quantify user outcomes. Advances in generative artificial intelligence may also enable more adaptive and natural conversational experiences, although future work must carefully address issues related to reliability, transparency, and user safety. Longitudinal evaluations will also be necessary to better understand sustained engagement and the long-term role of conversational support systems in promoting independent living and emotional well-being. Collectively, these directions highlight the importance of integrating empathic conversational design and accessibility-oriented interaction strategies in the development of digital support tools for older adults.

## 6. Conclusions

This paper presented UjaBienestar, a multimodal conversational chatbot specifically designed to provide emotional support, daily assistance, and accessible interaction for older adults. The proposed system integrates text- and voice-based communication, reminder functionalities, and empathic conversational design principles tailored to the needs of ageing users.

The preliminary pilot evaluation suggests that the system constitutes a preliminary exploratory conversational assistive technology approach for supporting accessibility, perceived emotional well-being, and everyday assistance among older adults. In particular, the findings highlight the relevance of multimodal interaction and emotionally supportive dialogue strategies as key factors for user acceptance and perceived usefulness. However, these findings should be interpreted as subjective user perceptions within a controlled pilot context.

Beyond its functional capabilities, the system was positively perceived as a form of companionship and emotional accompaniment, reinforcing the potential of assistive conversational systems as a promising preliminary social technology for healthy ageing.

Overall, this work contributes to the fields of human–computer interaction, assistive conversational systems, and digital support systems for older adults by providing an integrated framework that combines usability, accessibility and emotional support in a single solution.

## Figures and Tables

**Table 1:** Comparison between the proposed framework and representative conversational systems. *Note:* The comparison is intended as a high-level conceptual overview of representative functionalities and design orientations rather than as a benchmark performance evaluation.

System	Emotional Support	Voice Interaction	Accessibility-Oriented Design	Reminder Functions	Older Adult Focus	Multimodal
Alexa	Partial	Yes	Limited	Yes	No	Yes
Google Assistant	Partial	Yes	Limited	Yes	No	Yes
Replika	Yes	Partial	No	No	No	Partial
ElliQ	Yes	Yes	Yes	Partial	Yes	Yes
Woebot	Yes	No	Limited	No	No	No
Proposed System	Yes	Yes	Yes	Yes	Yes	Yes

**Table 2:** Summary of key usability, emotional support, and interaction findings from the pilot usability evaluation (n = 10).

Dimension	Main Finding	Frequency
Usability	Easy-to-use and intuitive interface	9/10
Voice interaction	Positively valued multimodal voice support	9/10
Emotional support	Reported feeling listened to/accompanied	10/10
Interaction barriers	Initial difficulties with onboarding/first use	2/10
Improvement requests	More natural responses and additional topics	7/10

## Data Availability

Restrictions apply to the datasets. The datasets presented in this article are not readily available due to privacy considerations related to participant interaction data collected during the pilot study.

## Appendix A. Conversation Transcriptions

**Figure 2: Interaction examples**

(a) Chatbot: “How can I help you today? Take your time to tell me what you need.”

(b) User: “It’s just that my son hasn’t come to see me for a long time, so I don’t know… I feel lonely.”

Chatbot: “I see… It’s hard when our children cannot visit us as often as we would like. You feel that emptiness, don’t you? Have you talked to him recently? Have you spoken with him by phone or in some other way? Sometimes, even if it’s not the same as a hug, staying in touch helps a lot. Tell me more if you’d like, don’t worry about bothering me. I’m here to listen to you.”

(c) User: “Remind me to take Paracetamol on Mondays, starting at 10 in the morning, every 12 h.”

Chatbot: “Until when? (e.g., 30 August)”

User: “2 September”

Chatbot: “Okay, perfect. I will remind you to take Paracetamol on Mondays, starting next Monday at 10 a.m., every 12 h, until 2 September. Does that sound good? Is there anything else I can help you with?”

**Figure 3: Interface transcription**

(a) Welcome screen: “Welcome to UjaBienestar!”

Subtitle: “Your university virtual assistant. Log in with your Google account to get started.”

Privacy message: “Your data is protected and is only used to personalize your experience.”

Features: “Smart Chat”, “Natural Voice”, “Reminders”

Button: “Continue with Google”

Footer: “University of Jaén—Virtual assistant for the well-being of older adults”

(b) Main interface:

User profile: “Michel”

Options: “Medications”, “Emergency”, “Log out”

System message: “Oh, Michel, you’ve got quite the attitude! I’m really glad to see you so willing to talk to me. But, honestly, the connection is still a bit… rebellious! It’s as if we were trying to communicate through an old telephone, with lots of interference.”

Top option: “Change character”

Input field: “Type a message…”

Interface elements: voice input button and send button.
